# An analysis of sustainability and performance indicators in Eco-Conscious trainers’ brands

**DOI:** 10.1038/s41598-025-04186-y

**Published:** 2025-07-01

**Authors:** María José Munguía Romero, Veronika Kapsali, Li Wang, James JC Busfield

**Affiliations:** 1 School of Engineering and Materials Science, Queen Mary University of London, Mile End Road, London, E1 4 NS UK; 2 London College of Fashion, University of the Arts London, London, SW1P 4 JU UK

**Keywords:** Sustainable fashion, Footwear and apparel, Trainers, Footwear, Sustainability, Sustainability challenges, Sustainability, Environmental impact

## Abstract

The fashion industry is known for its negative environmental and social impacts globally. Sustainability has become a priority for governmental agendas and concerned consumers. However, achieving sustainability remains a challenge for the industry due to its complex supply chains and systems. Specifically, the footwear industry accounts for 1.4% of global carbon emissions, with trainers being one of the most popular types of footwear among consumers. Recent studies have highlighted their ecological footprint, prompting companies to implement various actions to address this issue. Nevertheless, the environmental impact of this sector continues to pose significant challenges. This paper conducts a content analysis to examine and classify the actions taken by 13 Sustainable Trainers’ Brands according to five categories: Sustainable Materials, Sustainable Manufacturing, Sustainable Distribution, End-of-Life Recovery, and Eco-Design. Results show that Sustainable Materials is the most frequently adopted approach for sustainability, while Eco-Design is the least emphasised area. Additionally, the indicators used to assess sustainability in this sector primarily focus on the Environmental Sphere, with Sustainable Materials being the most prominently coded category. This study identifies Eco-Design as an area that requires further attention in future developments to address sustainability from a broader perspective.

**Supplementary Information:**

The online version contains supplementary material available at 10.1038/s41598-025-04186-y.

## Introduction

For this study, trainers will be defined as footwear used for sports activities and casual wear, primarily for aesthetic and comfort purposes. However, the term “Sneakers” is also commonly used to describe the same concept in the research field. Trainers are widely regarded as an accessory that most people have encountered, with a high likelihood of owning and wearing a pair^1^. According to a report by Brand Essence^2^, trainers are the preferred footwear among young people, with running shoes dominating the market due to the rising popularity of sports and outdoor activities. Today, Nike’s Air Jordan is no longer considered merely a basketball shoe but a fashion statement^3^.

A 2022 report by Mintel, a global market research agency, revealed that 77% of consumers in the USA purchased at least one pair of trainers within a year, while 29% bought more than three pairs during the same period^4^. On a global scale, 1.2 billion pairs of trainers were sold in 2022, a figure projected to increase to 1.4 billion by 2028^5^. Over the past few decades, trainer consumption has increasingly been linked to social and cultural purposes, such as fashion trends and self-expression. This shift has reshaped how trainers are perceived and purchased^1,3,6–8^.

The expansion of fast fashion has significantly increased the consumption of fashion goods, thereby amplified their environmental footprint and contributed to greater ecological impact and waste^9^. The high demand and consumption of trainers have directly influenced the environment, resulting in negative ecological effects.

The footwear industry alone accounts for 1.4% of global carbon emissions^10^, representing one-fifth of the fashion industry’s total emissions, excluding the use phase of these products^9^. The manufacturing process for trainers generates approximately 313 million metric tons of carbon dioxide annually, equivalent to the emissions from 66 million cars^11^. A single pair of synthetic running shoes is estimated to generate 14 kg of CO₂ emissions, with most of the impact arising during the processing and manufacturing stages. Trainers are composed of numerous components and require an average of 360 steps to assemble, making their production energy-intensive and resource-demanding, thus increasing their environmental footprint across the supply chain^12^.

This study aims to provide a deeper understanding of the practices adopted by trainer brands to integrate sustainable development into their supply chain management. It suggests that environmental sustainability can be achieved not only through the use of sustainable materials but also via complementary practices. However, further research is needed. This work does not seek to compare brands but rather to analyse them collectively, identifying the areas currently being addressed and highlighting gaps that offer opportunities for sustainable development within the sector.

To assess the status of sustainability in the footwear sector, this research is guided by three Research Questions (RQ) RQ1: What aspects of sustainability are being addressed by sustainable trainer brands? RQ2- What tools are used to estimate the impact of these aspects? RQ3- What steps could be taken to surpass the current boundaries and limitations?

The study begins with an introduction to the growing importance of trainers, the environmental impacts of their production and disposal, and the role of sustainability in this sector. It then outlines content analysis as the chosen research methodology, introducing the list of brands selected for the study and the criteria used for their categorisation. The subsequent sections present the results, discuss the findings and conclusions, and propose approaches for further research.

## Literature review

The footwear and apparel industries are significant consumers of water and energy and are major contributors to microplastic pollution^13^. The high volume of products manufactured, a complex logistics network, unsustainable production processes, and increasing textile waste render the fashion supply chain a threat to the environment^9,14^. Consequently, sustainability has become a critical issue to address in this sector^15^.

For trainers, materials used in manufacturing account for an average of 20% of their total carbon footprint^11^, with many being derived from fossil fuels. Petroleum-based materials are energy-intensive, while natural materials often require large quantities of water and pesticides^16^. Manufacturing processes represent the highest percentage of a trainer’s carbon footprint^11^ due to the extensive operations involved in production.

In the USA alone, an estimated 300 million pairs of trainers are discarded annually, with 95% ending up in landfills^17^. Depending on the materials used, their degradation time ranges from 40 to 1,000 years^11^. Additionally, fewer than 5% of trainers are recycled at the end of their life^18^. This low recyclability is due to their complex design, the use of petroleum-based components, and energy-intensive manufacturing processes, all of which make trainers an environmental risk^18^.

In recent years, sustainability has gained prominence among fashion retailers worldwide^19^. Business performance and legislation are key drivers for environmental practices^20^ prompting companies to implement greener solutions^21^. Fashion and textile companies are increasingly integrating sustainability across their supply chains, from the design and development stage to working conditions and supplier standards, while also making this information accessible to consumers^19^. Young adult consumers recognise that sustainability is an important factor in the manufacturing of fashion goods that can influence their decision-making process when buying^22^, demanding clearer and transparent information about the products they consume. Although sustainable development has been acknowledged, a deeper understanding of the effectiveness of these practices is needed^21^.

Sustainability must play a significant role in business decision-making, particularly as current legislation and regulations come into force^23^. Achieving sustainability requires consideration at the managerial level, not just in design and manufacturing^21^. While corporations have the capacity to drive change, they must be held accountable for their impact^15^. Assessing the environmental, economic, and social consequences of their decisions obligates decision-makers to address these challenges^24^.

Integrated decision-making, which considers environmental, social, and economic goals simultaneously, enables decision-makers to identify issues and evaluate criteria effectively^24^. Developing suitable approaches to sustainability requires a deeper understanding of the actors influencing business behaviour^20^. While decision-making does not explicitly define how to act under uncertainty, without integration, businesses cannot achieve sustainable development^24^. In practice, the environmental dimension of sustainability has become the primary focus for addressing sustainable development, though few studies have examined how supply chain management is pursued in the fashion sector^21^.

Organizations and programmes such as the Waste Prevention Programme for England^25^, the EU Strategy for Sustainable and Circular Textiles^26^, the UN Alliance for Sustainable Fashion^13^, and the Ellen MacArthur Foundation^27^ are working to mitigate the environmental impact of the fashion industry. They do so by introducing schemes that apply circular economy principles to reduce the sector’s carbon footprint. Fashion companies are adopting diverse strategies, including the use of cleaner energy, improved worker conditions, responsibly sourced materials, cleaner manufacturing processes, and end-of-life recovery solutions^28^. In addition, apparel brands are exploring reselling, renting, and repairing business models to transition to more sustainable practices^29^. From a design perspective, circular strategies are being pursued, such as selecting sustainable materials, promoting product longevity, designing for timeless appeal, minimising waste, and enhancing repairability and recyclability^30^. Furthermore, innovation in design techniques and environmental education for consumers are necessary to encourage more responsible choices^28^.

Many companies have been influenced by transparency pledges to disclose their social and environmental impact^31^, fostering positive reactions from consumers and investors^32^. However, there remains a disconnect between corporate commitments, the systems used to measure them, and the practices for reporting results. This gap complicates efforts to understand and evaluate the effectiveness of sustainability initiatives^33^.

While a clearer understanding of corporate sustainability performance is essential, researchers have questioned how much sustainability data influences decision-making within companies^34^. Studies of popular trainer brands suggest that the footwear industry’s progress in sustainable development lags behind other sectors^35^, indicating opportunities for future improvements^18^. However, the primary barrier to sustainability is a lack of knowledge about how to achieve it^36^. This highlights the need for further research into sustainable practices, the decision-making processes behind them, and the indicators used to measure their effectiveness.

### Research method

A content analysis was conducted using NVivo 12, a qualitative analysis tool, to examine the sustainability approaches and indicators employed by sustainable trainer brands. Content analysis provides both quantitative and qualitative insights^37^ by transforming large qualitative datasets into quantifiable results^38^ through the categorisation of content^39^. This methodology involves identifying relevant sentences that meet specific criteria and coding them according to a selected quality assessment scale^40^. It is a widely used research method for evaluating organisations’ social and environmental disclosures^37^, making it well-suited for this study.

### Scope and methodology

The first step in the study was to define its scope. From a sustainability perspective, companies were categorised into two main groups. The first group, referred to as Group A, consists of corporations that are unconcerned about sustainability and Group B, which consists of companies implementing sustainability practices within their operations through tools and strategies such as standards, social and environmental audits, and fair practices^15^ Only brands that fell under the second category (Group B) were included in the study. An online search using Google was conducted to identify suitable brands. Keywords such as “Sustainable Trainers’ Brands,” “Sustainable Trainers,” and “Sustainable Sneakers” were used for the search. The initial search yielded 120 brands. To refine this list, the following exclusion criteria were applied:

- **Product Focus**: Brands producing boots, sandals, loafers, or other types of footwear besides trainers were excluded to maintain focus on trainer-specific footwear.
- **Luxury Brands**: Brands characterised by high prices, exclusivity, and aspirational attributes were excluded, as these products are not accessible to the majority of consumers^41^.
- **Multi-Category Brands**: Brands manufacturing apparel, accessories, or other products alongside trainers were excluded to avoid confusion and ensure clarity regarding sustainability claims specific to trainer production.
- **Sustainability Information**: Brands without a dedicated sustainability section on their websites were excluded to meet the study’s initial criteria.
- **Language Accessibility**: Brands with websites not available in English or without English translations were omitted to prevent misinterpretation due to language barriers.

After applying these criteria, the list was narrowed to 13 brands: Bahe, Clae, Ethletic, Flamingos Life, Orba, Sans Matin, Saola, Sole Rebels, Thousand Fell, Waes, Womsh, Zeta, and Veja. Table 1 presents the final list of brands, and the acronyms used for their identification throughout the study.

**Table 1:** List of sustainable trainers’ brands analysed for the study.

Brand	Acronym	Company size	Location
Bahe	BH	Micro	London, United Kingdom
Clae	CL	Small	Los Angeles, US
Ethletic	ET	Small	Lübeck, Germany
Flamingos life	FL	Small	Alicante, Spain
Orba	OR	Small	Wellington, New Zealand
Sans matin	SM	Micro	London, United Kingdom
Saola	SL	Micro	Haute-Savoie, France
Sole rebels	SR	Medium	Addis Ababa, Ethiopia
Thousand fell	TF	Micro	New York, US
Waes	WS	Micro	Kingsbridge, United Kingdom
Womsh	WM	Small	Vigonza, Italy
Zeta	ZT	Micro	Bordeaux, France
Veja	VJ	Large	Paris, France

Enlists the names of the trainers’ brands analysed for this study, followed by an acronym used to identify them in the results.

Brands’ company size was determined according to the European Union SME definition^42^ and classified based on the number of employees stated on the webpage and LinkedIn website of each brand.

The aim was to extract all publicly available information regarding the brands’ sustainability commitments and classify it to identify the areas being addressed and the indicators used to evaluate those areas. To develop the analysis only information from the brands’ websites was reviewed, focusing on sections related to sustainability and company history. Sustainability information was found under various headings, such as “Fair for All,” “Project,” “Our Commitments,” “Manifesto,” “Impact,” “Our Ethos,” “Mission,” “Eco-Conception,” and “Materials.” Additional sections like “Innovation,” “Our Workshops,” “Our Manufacturers,” “Our Charities,” “Biodiversity,” and “Materials” were consulted to support and validate the information provided in the sustainability sections. To verify materials-related claims, a randomly selected pair of trainers from each website was examined.

### Data preparation

To facilitate the analysis, relevant website sections were saved as PDFs. For websites with interactive interfaces that hindered direct saving, dropdown texts were exported into Word files and then converted to PDFs. Sections from the same brand were combined into a single PDF file, resulting in 13 brand-specific PDF documents.

These PDF files were then uploaded into NVivo software for coding and classification, enabling a systematic analysis of each brand’s sustainability approaches and indicators. The analysis assessed and classified the data according to the codes and sub-codes established for each of the three stages of the study: Stage 1-Triple Bottom Line (TBL) Classification, Stage 2-Environmental Sphere Focus and Stage 3- Indicators Framework.

### Coding stages

The first stage categorised the actions based on the Triple Bottom Line (TBL) framework, which encompasses the Environmental, Economic, and Social Spheres of sustainability ³⁷. The Social Sphere sub-codes are focused on the workers’ rights and well-being. The Economic Sphere sub-codes target Transparency, Traceability and Profit. While the Environmental Sphere sub-codes align with the stages of the fashion supply chain.

**Fig. 1:**
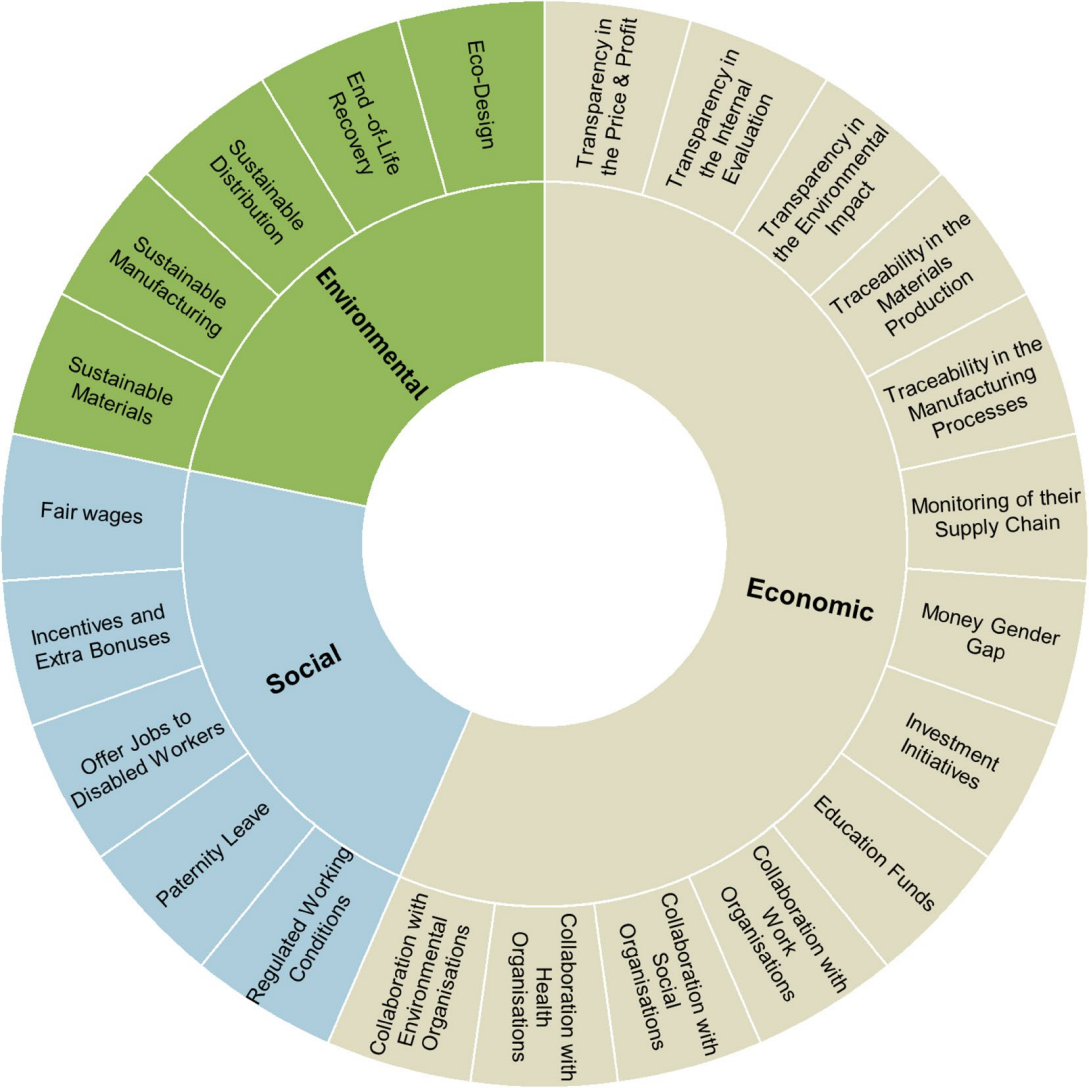
TBL Coding Framework indicates the sub-codes encompassed in the Environmental, Economic and Social spheres, respectively, for this study.

Additional codes were created during the analysis to address new issues as they emerged. The **TBL Coding Framework**, including its codes and sub-codes, is illustrated in Fig. 1.

This coding approach provided a structured understanding of the various sustainability strategies employed by trainer brands, offering a comprehensive overview of how sustainable development practices are integrated across their supply chains. Notably, to prevent double counting, codes and sub-codes mentioned multiple times within the same brand were only recorded once.

**Fig. 2:**
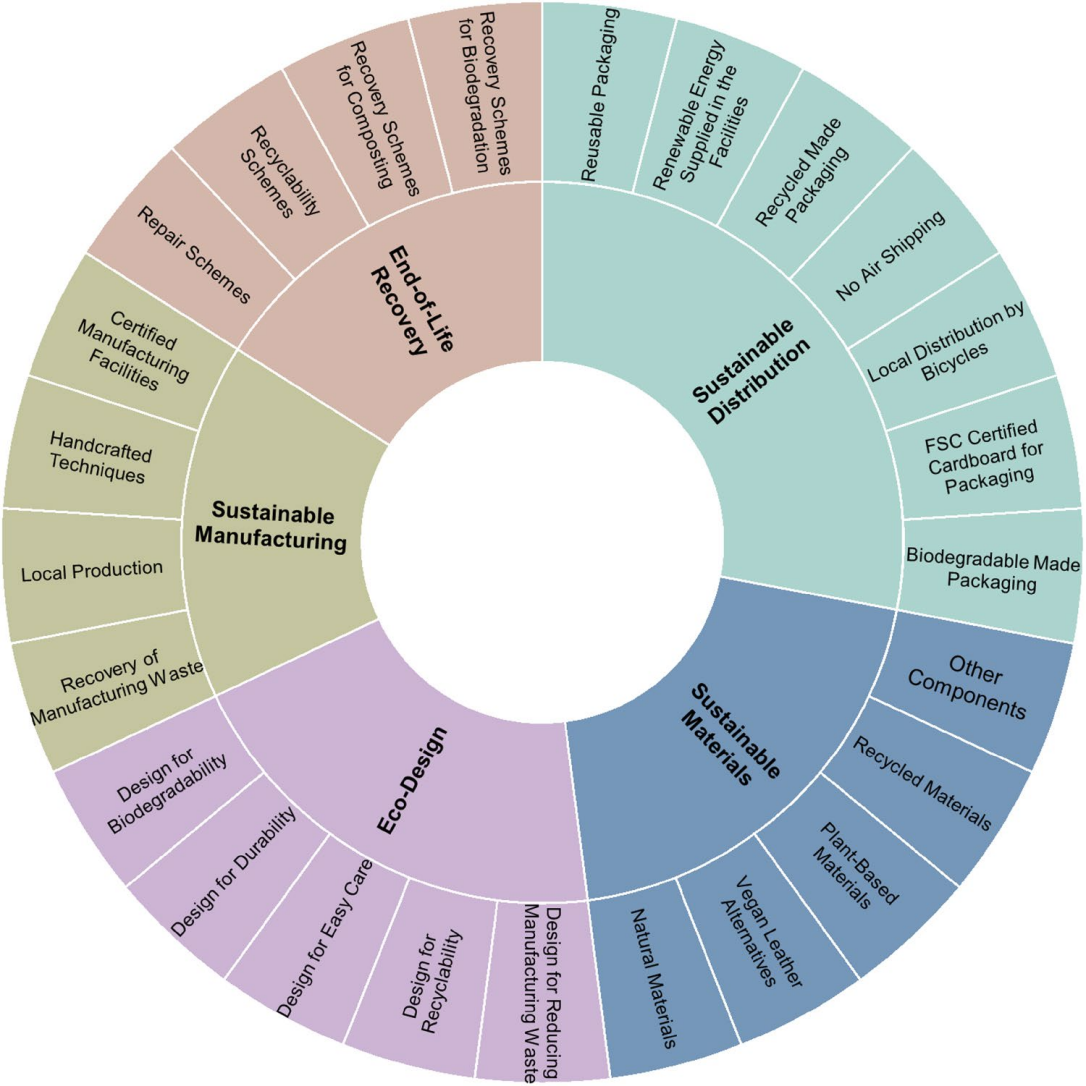
Environmental Sphere coding framework. shows the sub-codes encompassed in the Environmental Sphere, determined for this study. The 5 sub-codes are: Eco-Design, Sustainable Materials, Sustainable Manufacturing, Sustainable Distribution and End of Life Recovery. The image indicates the sub-codes encompassed in these 5 previously mentioned areas.

The second stage concentrated on the Environmental Sphere and its sub-areas. The corresponding Environmental Sphere Coding Framework, with its associated codes and sub-codes, is depicted in Fig. 2. Detailed definitions of these sub-codes are provided in the Code Book included in the Appendix.

In the third stage, the analysis identified the indicators used to measure the environmental, economic, and social impacts within the sustainable trainers’ supply chains. The Coding Indicators Framework addressed seven sub-codes representing areas assessed by the indicators: Social, Economic, Economic and Environmental, Economic and Social, Environmental, Environmental and Social, and Environmental Social and Economic.

This coding framework, encompassing all relevant codes and sub-codes, served as the baseline classification for the study. The initial results are presented as an aggregate; however, individual brand-specific findings are also included. Each subcode’s representation was calculated as a percentage of the total number of actions applied by each company, providing a quantitative perspective on their sustainability efforts.

The analysis and results are presented in the following sections. The study’s findings offer valuable insights into the current state of sustainable trainers’ brands and highlight key areas that require attention in future efforts to enhance sustainability within the sector.

## Results and discussion

### Stage 1- Triple Bottom Line (TBL) classification

Research indicates that fashion brands have predominantly focused on the Environmental Sphere of sustainability^43^. However, independently assessing the three spheres of sustainability—Environmental, Economic, and Social—limits the effectiveness of overall sustainability performance^21^. The results of the analysis reveal that the Environmental Sphere has the highest coding recurrence among sustainable trainers’ brands, with a 65% of coverage compared to the other two spheres of the Triple Bottom Line (TBL) framework, Economic Sphere 27% and Social Sphere 8% (see Table 2). For sustainable practices to be effective, all three spheres must be addressed holistically to avoid overemphasising one at the expense of the others^15^. Improvements are therefore required across the Environmental, Economic, and Social dimensions for fashion brands to achieve meaningful progress in sustainability^44^. That said, it has been argued that a company’s environmental stance often shapes the prioritisation of TBL elements in its decision-making processes^45^.

**Table 2:** Results of the TBL coding framework.

TBL sphere	Sub-codes	TBL coverage
Economic	Collaboration with environmental organisations	27%
	Collaboration with health organisations	
	Collaboration with social organisations	
	Collaboration with work organisations	
	Education funds	
	Investment initiatives	
	Money gender gap	
	Monitoring of their supply chain	
	Traceability in the manufacturing processes	
	Traceability in the materials production	
	Transparency in the environmental impact	
	Transparency in the internal evaluation	
	Transparency in the price & profit	
Environmental	Eco-Design	65%
	End-of-life recovery	
	Sustainable distribution	
	Sustainable manufacturing	
	Sustainable materials	
Social	Fair wages	8%
	Incentives and extra bonuses	
	Offer Jobs to disabled workers	
	Paternity leave	
	Regulated working conditions	

Shows the sub-codes encompassed in the environmental sphere, determined for this study. The 5 sub-codes are: Eco-Design, Sustainable Materials, Sustainable Manufacturing, Sustainable Distribution and End of Life Recovery. The image indicates the sub-codes encompassed in these 5 previously mentioned areas. .

**Fig. 3:**
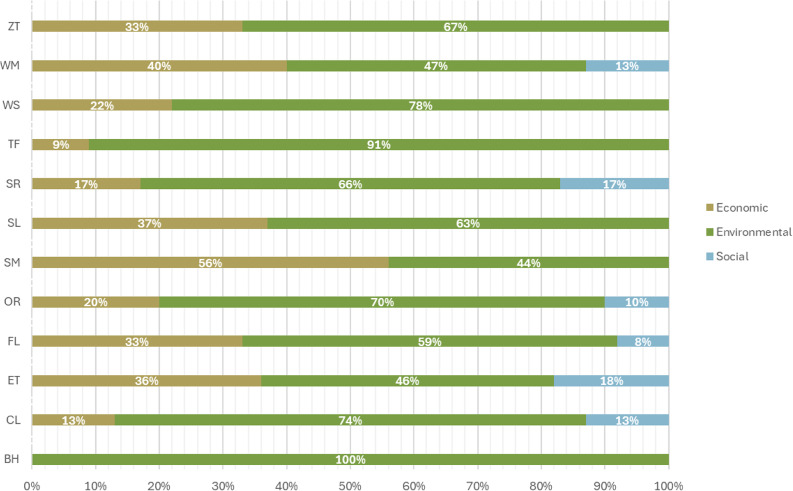
Results of the TBL Spheres coding, by brand indicates the results of the TBL Coding Framework per brand. It shows the recurrence of the Spheres on each brand and colour codes the Environmental Sphere as green, Economic Sphere as beige and Social Sphere as blue.

Ideally, all spheres should be considered and positively impacted. However, as shown in Fig. 3, the Environmental Sphere is acknowledged by all brands, with representation ranging from a minimum of 44% to a maximum of 100%. The Economic Sphere is present in 12 of the 13 brands, with representation varying between 9% and 56%. The Social Sphere is the least represented, appearing in only 7 of the 13 brands, with a minimum representation of 8% and a maximum of 18%. Additionally, it can be observed that small, medium and large companies analysed for this study acknowledge the 3 TBL Spheres. In contrast, micro-size companies tend to focus only on 2, the Environmental and Economic Spheres. Regarding business location, the tendency shows that Sustainable Trainers’ Brands are primarily located in Europe and the United Kingdom.

Environmental issues are often region-specific^24^ and largely driven by regulations, which serve as key catalysts for the development of sustainable practices^20^. These factors make environmental matters highly relevant to both the public and private sectors. This influence is evident in the TBL Coding Framework, where the Environmental Sphere’s high recurrence can be attributed to the impact of environmental regulations, which often drive industrial change.

While legislation provides a bridge between societal needs and business interests, it is not the sole driver of sustainable development and cannot achieve success on its own^20^. Identifying the sustainable practices applied in fashion supply chain management motivates companies to adopt the most appropriate approaches for their operations^21^. This enhances decision-making processes and encourages the adoption of more suitable approaches based on prior experience^21^. Effective decision-making is critical for sustainable development as it forms the foundation for addressing sustainability principles^24^.

Understanding companies’ approaches to sustainability and identifying key elements in this field can facilitate the integration of sustainable practices^34^.

## Stage 2- Environmental Sphere focus

Given that the Environmental Sphere has the highest coding frequency and the most available information, the subsequent analysis focuses on this sphere. The results of the sub-code analysis within the Environmental Sphere, shown in Table 3, reveal that Sustainable Materials rank highest, being coded 72 times with a coverage of 64%. This is followed by Sustainable Distribution 12%, End-of-Life Recovery 9%, Sustainable Manufacturing 8%, and Eco-Design 7%.

**Table 3:** Environmental Sphere coding results for all brands.

Environmental sphere coding framework	Sub-codes	Coverage
Eco-design	Design for biodegradability	7%
	Design for durability	
	Design for easy care	
	Design for recyclability	
	Design for reducing manufacturing waste	
End-of-life recovery	Recovery schemes for biodegradation	9%
	Recovery schemes for composting	
	Recyclability schemes	
	Repair schemes	
Sustainable distribution	Biodegradable made packaging	12%
	FSc certified cardboard for packaging	
	Local distribution by bicycles	
	No air shipping	
	Recycled made packaging	
	Renewable energy supplied in the facilities	
	Reusable packaging	
Sustainable manufacturing	Certified manufacturing facilities	8%
	Handcrafted techniques	
	Local production	
	Recovery of manufacturing waste	
Sustainable materials	Natural materials	64%
	Vegan leather alternatives	
	Plant-based materials	
	Recycled materials	
	Other components	

shows the results of the environmental sphere coding framework, indicating the recurrency of appearance of the sub-codes encompassed in the areas of eco-design, sustainable materials, sustainable manufacturing, sustainable distribution and end of life recovery colour coding them as lilac, navy blue, olive green, aqua blue and pink respectively. results considered all the analysed brands as a set and are expressed in percentages. .

**Fig. 4:**
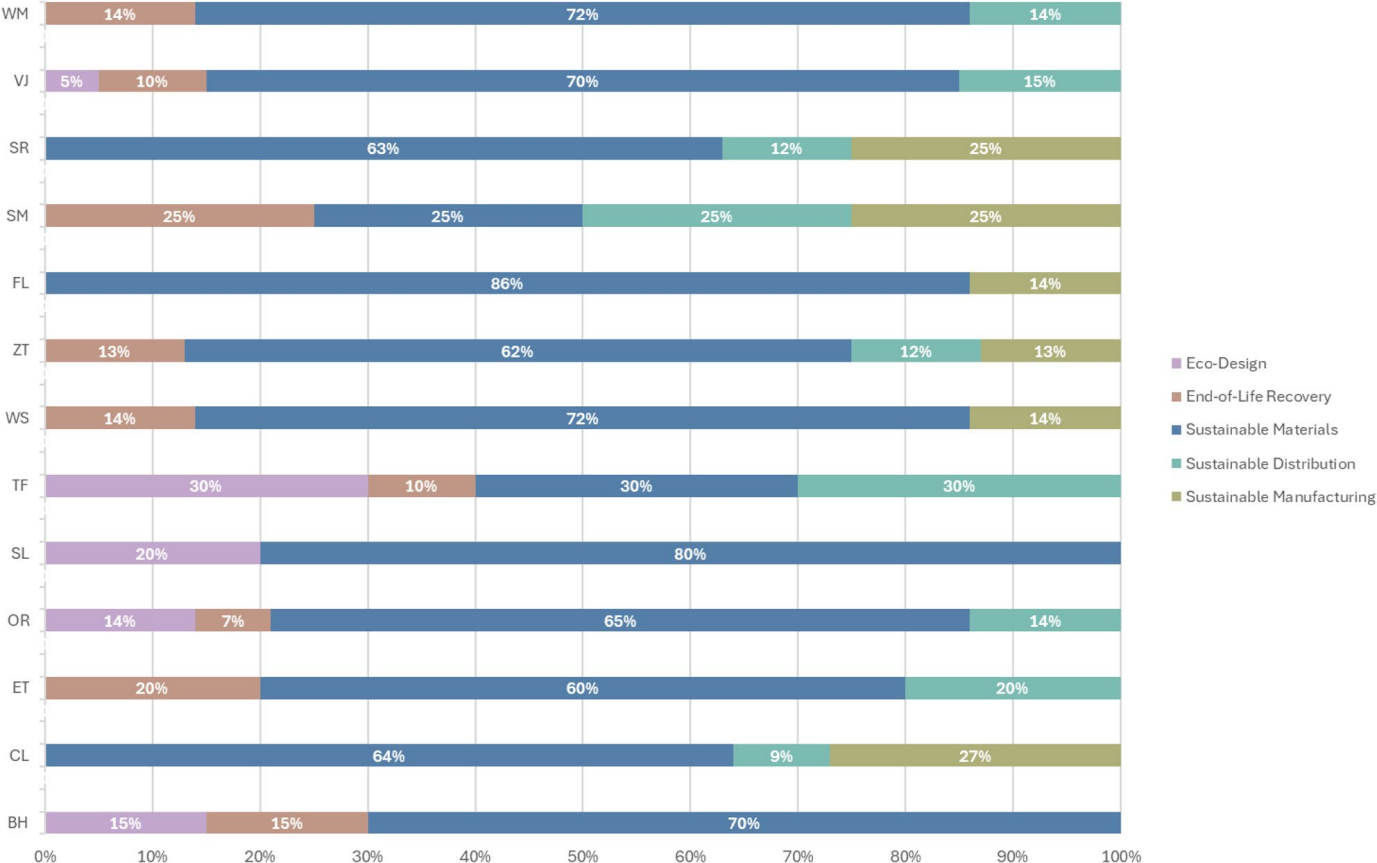
Results of the TBL Spheres coding, by brand indicates the results of the Environmental Sphere Coding Framework per brand. It shows the recurrence of the sub-codes for each area and colour codes the Eco-Design lilac, Sustainable Materials navy blue, Sustainable Manufacturing olive green, Sustainable Distribution aqua blue and End of Life Recovery pink.

Furthermore, the results presented in Fig. 4 indicate that Sustainable Materials is the only sub-code consistently recurring across all brands, with a maximum representation of 86% and a minimum of 25%. Sustainable Distribution is identified in 9 of the 13 brands, with representation ranging from a high of 30% to a low of 9%. Similarly, End-of-Life Recovery appears in 9 brands, with the highest percentage at 25% and the lowest at 7%. In contrast, Sustainable Manufacturing is referenced by only 6 brands, with a maximum representation of 27% and a minimum of 13%. Lastly, Eco-Design is the least recurring sub-code, appearing in just 5 brands, with representation ranging from a high of 30% to a low of 5%.

Environmental protection often focuses on specific natural resources, such as water and air, without adequately considering other elements that influence ecological well-being^24^. This limited perspective makes the assessment of the Environmental Sphere and its associated practices uneven and inconsistent. Although companies are attempting to adopt more sustainable practices, sustainability remains a challenge for fashion and footwear businesses. Efforts to implement sustainable development solutions frequently face economic challenges, as innovative strategies often entail higher costs^9^. While sustainable practices have already generated economic benefits^20^, their introduction can also increase operating expenses^45^. Moreover, sustainable performance may vary with company size, as detailed environmental impact assessment systems are more difficult to apply in small businesses^21^. The integration of sustainable practices within an organization can also be hindered when implemented at a low level, making it difficult for stakeholders to engage and contribute effectively^24^. Research shows that for sustainable practices to succeed, all stages of a product’s life cycle must be considered and integrated^46^.

### Stage 3- Indicators Framework

The results of this analysis have revealed a wide range of practices applied to the Environmental Sphere. However, these strategies are often introduced independently, potentially compromising their overall effectiveness. A clearer understanding of the connections between these practices could create a more cohesive approach to addressing environmental impacts at every stage.

To gain a deeper understanding of how Sustainability Indicators work, the Stage 3 coding phase of this analysis focused on the Sustainability Indicators used by Sustainable Trainers’ Brands. Results show that the Environmental Sphere, already the most coded, also has the highest number of indicators, with 20 indicators coded 41 times with a coverage of 65%. This is followed by Social Indicators, with 11 indicators coded 12 times and a coverage of 19%; Environmental and Social Indicators combined, with 6 indicators coded 6 times with a coverage of 10%; and Environmental, Social, and Economic Indicators together, with 2 indicators coded 4 times with a coverage of 6% (see Table 4).

These results highlight the disparity in the number of indicators for each sphere, reflecting the conclusions drawn from the first coding. Indicators predominantly target the Environmental Sphere, demonstrating the uneven attention given to each TBL dimension and revealing the Economic Sphere as lacking indicators altogether. Economic indicators such as GDP do not integrate the environmental and social effects of economic activities, indicating a gap in developing tools that capture the interplay between the economy and the environment^24^. This gap underscores the need for further development.

**Fig. 5:**
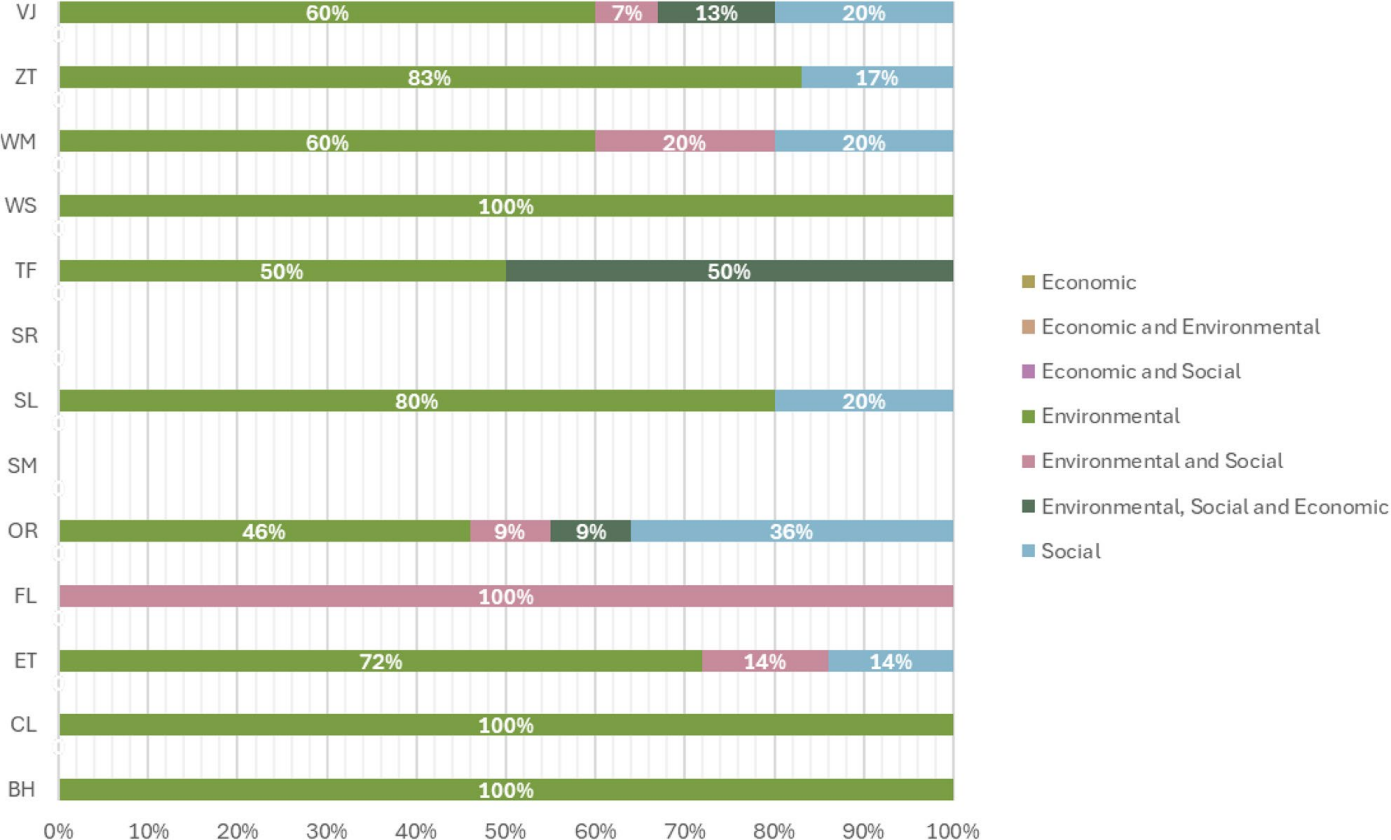
Classification of the environmental indicators according to the sub-codes of the environmental sphere. Environmental sphere coding results for all brands indicates the results of the Environmental Sphere Coding Framework per brand. It shows the recurrence of the sub-codes for each area and colour codes the Eco-Design lilac, Sustainable Materials navy blue, Sustainable Manufacturing olive green, Sustainable Distribution aqua blue and End of Life Recovery pink.

It was also observed that 10 out of the 13 brands apply indicators to assess the Environmental Sphere of the TBL, while 6 out of the 13 brands use indicators to evaluate the Social Sphere (see Fig. 5). Additionally, 2 of the analysed brands do not mention any indicators in their publicly available information.

**Table 4:** Results of the sustainability indicators analysis by brand.

Coding Indicators Areas	Indicators	Coverage
Environmental	CO2 emission assessment	65%
	Ecologi-zero impact	
	GHG emission assessment	
	P4 F	
	ISO 14,001	
	LCA	
	ASTM D 6400	
	LWG certified	
	EN 13,432	
	Fairtrade cotton	
	FSC certified	
	GOTS certified	
	GRS certified	
	ISO14855-1	
	Oeko-tex certified	
	Organic traced leather certified	
	PETA approved vegan	
	ROA certified	
	USDA organic	
	Veja restricted substances policy	
Environmental and Social	Bcome	10%
	Cradle to cradle	
	Fairtrade ctandard	
	Renoon	
	Tracycle	
	Womsh ethical code	
Environmental, Social, Economic	B corp certified	6%
	Fair for life certified	
Social	BSCI	19%
	Ethical trade audit	
	Fairtrade principles	
	Global living wage	
	International labour standards	
	ISO 45,001	
	Orba flexible framework	
	Orba supplier code of conduct	
	Orba supplier risk assessment	
	SA8000	
	Veja code of conduct	

indicates the Sustainability Indicators encountered in the analysis. Results are analysed as a set from all brands and are indicated in percentages according to their coverage. Indicators assessing the Environmental Sphere are colour coded as green, Indicators assessing the Social Sphere are colour coded as blue, Indicators assessing the Environmental and Social Sphere are colour coded as pink, and Indicators assessing the Environmental, Social and Economic Sphere are colour coded as dark green.

Information about a company’s sustainability performance can be shared both externally and internally, influencing the decision-making of stakeholders throughout and beyond the organization^34^. To achieve this, reliable data on the consequences of poor environmental, social, and economic practices is essential. Indicators that periodically report achievements can guide decision-makers in developing effective policies^34,45^. Establishing Sustainability Indicators begins with identifying priorities. Doing so provides a clearer starting point in the transition toward more sustainable practices^47^.

Using indicators at early stages can provide valuable information about the consequences of decisions made throughout the supply chain. Because companies currently define their own priorities and influence the development of their Sustainability Indicators, the concept of sustainability varies between organizations. However, companies within the same sector often share similar concerns^34^. Without indicators, it is difficult to measure improvements in a company’s sustainability performance^34^.

The development of standardized indicators poses a dilemma, as they must adapt to different geographical regions, cultures, values, objectives, and targets^34^. Indicators enable progress tracking and help justify choices made across the supply chain^45^. They are key to achieving sustainability because they challenge business practices and have the potential to drive change^48^. Still, using indicators to measure the effectiveness of businesses’ approaches remains challenging^49^. Without indicators that measure the progress and effectiveness of actions and methods aimed at reducing the environmental footprint of products like trainers, these challenges remain unresolved.

A wide range of practices suggests that, in reality, there is currently no clearly defined path to address sustainability in the trainers’ sector. The available indicators are influenced by existing processes not suitable for every company, making sustainability assessments more difficult^34^. Despite a large number of indicators and standards, the need for clear guidelines and frameworks remains a challenge due to limited insight and guidance on current performance^15^. Existing standards and indicators use different criteria to determine their effectiveness, resulting in ambiguity and uncertainty that complicate decision-making^15^. Indicators remain questionable due to a lack of validity and consensus^50^, and they have not provided a unified view of Sustainable Development or an integrated perspective on sustainable practices^15^.

Coherence and consistency among decision-makers are needed^24^ to effectively address Sustainable Development. However, each business has different processes, and not all companies are at the same level of sustainability maturity^34^. Without a standardized framework for sustainability, companies may feel less pressure to improve, thus hindering the establishment of collective standards and recognized Key Performance Indicators (KPIs).

The final analysis of the study connects the Environmental Indicators identified during the analysis with the Environmental Sphere’s sub-codes, illustrating which sub-codes each indicator assesses (see Table 5). The results show that indicators evaluate only the Sustainable Materials and Sustainable Manufacturing sub-codes. Among these, Sustainable Materials remains the most frequently assessed area, with 14 indicators, followed by Sustainable Manufacturing with 6 indicators. This finding aligns with previous observations that there is no established framework to systematically assess sustainability in the trainers’ sector.

**Table 5:** Classification of the environmental indicators according to the sub-codes of the Environmental Sphere.

Indicator	Eco- design	End of life covery	Sustainable distribution	Sustainable manufacturing	Sustainable materials
CO2 emission assessment				X	
Ecologi-zeroimpact				X	
GHG emission assessment				X	
P4 F					X
ISO 14,001				X	
LCA				X	
ASTM D 6400					X
LWG certified					X
EN 13,432					X
Fairtrade cotton					X
FSC certified					X
GOTS certified					X
GRS certified					X
ISO14855-1					X
Oeko-tex certified					X
Organic traced leather certified					X
PETA approved vegan					X
ROA certified					X
USDA organic					X
Veja restricted substances policy				X	

enlists the indicators encountered in the study and indicates which of the 5 areas encompassed in the Environmental Sphere coding are being assessed by each indicator. The areas are colour coded as Eco-Design lilac, Sustainable Materials navy blue, Sustainable Manufacturing olive green, Sustainable Distribution aqua blue and End of Life Recovery pink.

Environmentally friendly materials can minimize the ecological impact of fashion goods^28^, yet material selection alone is not sufficient. Beyond materials, the production methods significantly influence a product’s environmental footprint, making manufacturing processes a primary source of energy consumption^9^. The high carbon emissions associated with trainers’ manufacturing^11^ and their low recyclability rate make manufacturing processes and recovery schemes key priorities in this sector. Decisions taken at the design stage can reduce the ecological footprint by altering production processes^24^, thereby decreasing product waste, the use of chemical substances, and water consumption and pollution^21^. Such design-led decision-making can enhance the overall sustainability performance of a product. Indeed, research has shown that fashion companies incorporating sustainable design principles achieve better environmental performance metrics than those that do not^21^.

Implementing circularity strategies, including improving product recyclability, can prevent waste from ending up in landfills and encourage the use of recovered materials. The current business model inherently produces waste, and the system must be redesigned to prevent it^9^. Improved communication between design and manufacturing can effectively reduce pre-consumer waste in production stages^51^, thereby mitigating the ecological impact of this phase. This can be accomplished through a deeper understanding of product construction and its manufacturing implications. For instance, in garment production, pre-consumer waste is influenced by the placement of design patterns on the fabric^9^, underscoring the critical role of design in waste reduction. While design can positively impact environmental performance throughout the supply chain, this analysis has identified relatively few strategies focused in this area.

Applying circular design principles at the early stages of product development can prevent and reduce textile waste throughout the product’s life cycle^28^, thereby minimizing the need to address it in later phases. According to the European Commission, more than 80% of a product’s environmental impact is determined at the design stage^52^. Despite this, the results still indicate that there is very limited adoption of Eco-Design approaches.

Literature highlights the positive outcomes of implementing sustainable design practices to improve sustainability metrics and reduce carbon footprints^53^, underscoring the importance of decision-making at the early stages of product development. However, these decisions are often made under conditions of uncertainty due to limited and ambiguous information about ecological consequences^45^. Additionally, research has shown that young consumers prefer sustainable products that do not compromise the aesthetics of the product^22^, which demonstrates why design needs to be prioritised in the making of sustainable products. In response, companies tend to adopt a set of simple rules for environmental decision-making and concentrate on measurable practices, hoping these efforts will indirectly benefit other areas they cannot directly control.

As a result, the environmental benefits of these principles often go unassessed and remain difficult to validate as genuinely effective^45^. Transparency plays a crucial role in driving sustainability; it fosters trust, educates consumers, and encourages them to make more informed decisions^28^. This openness can inspire positive responses from stakeholders and consumers alike^32^.

While decisions made at the design stage can positively influence the environmental performance of trainers, the study found no indicators dedicated to evaluating these design-stage decisions. Further research is needed to develop assessment methods that address sustainability from a more systematic perspective. Scholars have noted that sustainability indicators in the fashion and textile industry are narrowly defined and lack effectiveness due to inconsistent formats, concepts, and measurements. These discrepancies create gaps between sustainability goals and actions, resulting in an unclear understanding of the available information^19,33,48,49,54–57^.

Indicators can facilitate change by objectively assessing corporate sustainability performance and enabling companies to track costs while meeting regulatory requirements^20,55^. However, the wide variety of indicators currently in use hinders transparency and comprehension of their effectiveness.

The fashion industry’s complex and globalised logistics network exacerbates decision-making challenges in environmental matters by increasing uncertainty. This uncertainty arises from limited information and restricted stakeholder interactions^45^. Some businesses have begun to incorporate sustainability performance data into their decision-making and strategic planning processes, suggesting that decisions should be aligned with data collection^34^. Reviewing past data can also influence corporate decision-making^34^. However, without indicators capable of systematically assessing sustainability, these challenges remain unsolved, and the sector remains without standardisation. Reducing all options to a single universal tool that considers every aspect of sustainability may prevent decision-makers from exploring diverse strategies, potentially inhibiting the effectiveness of sustainable development^24^.

Companies advocating for Sustainable Development often work toward certain Operating Principles that guide their goals, although these principles are not strictly defined. To support these aims, companies develop Technical Principles, which act as decision-making standards. Technical Principles help eliminate uncertainty, provide a clear baseline, and facilitate the transition to greener practices^45^. Indicators are essential to defining the parameters of sustainable performance, thereby supporting decision-makers in evaluating outcomes^45^.

Operating Principles set the overarching objectives that guide a company, while Technical Principles can be applied at each stage of the process to accomplish these objectives^45^. Although the trainers’ sector offers a variety of Operating Principles, no Technical Principles were detected in this study. This absence suggests a need for systematic indicators to establish a baseline for achieving Operating Principles. Defining these parameters would simplify decision-making and enable measurable outcomes and progress tracking^45^.

For sustainable development to succeed, goals must be guided by specific objectives^24^. Though goals may evolve with technology and scientific knowledge, provisional or interim goals can serve as starting points on the path toward a targeted aim^24^.

Sustainability must be addressed from multiple angles to drive substantial change. The results of this analysis indicate a unidirectional approach to sustainability within the trainers’ industry, as the Environmental Sphere remains the most thoroughly explored. However, areas within this sphere—such as Eco-Design, Sustainable Manufacturing, and End-of-Life Recovery—are less well-developed. Eco-Design, a critical stage in creating less environmentally harmful products, is notably underrepresented among sustainable trainers’ brands.

Regulations can drive changes in product manufacturing’s environmental impact. For instance, the Eco-Design for Sustainable Products Regulation (ESPR) aims to introduce circularity into the European market by improving the durability, reusability, repairability, recyclability, and resource efficiency of products^58^. The uneven consideration of design, manufacturing, and end-of-life stages correlates with the absence of a unified approach that acknowledges every life cycle stage and all stakeholders in the decision-making processes.

While Sustainable Trainers Brands have made efforts to mitigate their products’ environmental, social, and economic impacts, further research is needed to develop tools and indicators that assess sustainability in a collaborative, integrated manner. By addressing concerns at the early design stages, it may be possible to fully integrate sustainability into the supply chain.

Integrated decision-making requires more than one tool aligned with a defined goal. However, identifying appropriate Sustainable Development goals represents the true catalyst for change^24^. The Sustainable Trainers’ Market shows the need for integrated Technical Principles that establish a baseline for achieving Operating Principles. Although companies promote their individual aims, the need for Technical Principles to assess these aims remains unclear.

### Limitations

The first challenge identified in this study was the lack of standardised terminology in sustainability communications. Brands often use different terms to describe the same concepts, which impedes transparency and clarity, underscoring the need for standardisation in sustainability language within the fashion sector.

Additionally, this study faced constraints related to data accessibility and the varying degrees of formality in sustainability reporting and data collection^34^. While the analysis relied on publicly available information, discrepancies may arise due to the gap between what is publicly disclosed and the internal data held by brands. Future research should involve collaboration with businesses to gain deeper insights and reduce inconsistencies in the findings.

## Conclusions

The analysis shows that the Environmental Sphere of the TBL framework is a strong asset in advancing sustainability within the trainers’ industry. Materials currently dominate as the primary approach to addressing sustainability, yet aspects such as Sustainable Manufacturing, End-of-Life Recovery, and Sustainable Distribution remain less explored.

Although existing measures are moving in the right direction, the design stage still offers considerable potential for improvement. Emerging research suggests that design decisions play a critical role in shaping environmental outcomes. This study addresses this gap by demonstrating the need for indicators that specifically assess Eco-Design principles, denoting that the true impact of design-related choices on a trainer’s environmental footprint remains largely untapped. Such indicators would allow sustainability considerations to be prioritised at a decision-making level.

Additional research into sustainability reporting guidelines and policies is necessary to mitigate corporate greenwashing^59^. Future studies should focus on developing metrics that measure the success or failure of design-level decisions, thereby supporting a more sustainable trainers’ industry. Ultimately, collaboration between businesses and scholars is essential for creating new indicators and tools. Involving brands in the development of strategies is crucial to understanding the challenges and constraints within the supply chain. The outcomes of this study will, it is hoped, inspire the creation of more comprehensive solutions in this sector.

This study proposes that future research should focus on understanding the impact of early design-stage decisions and developing indicators that assess sustainability from a unified perspective—one centred on Eco-Design principles and their connection to all stages of trainer manufacturing. Strengthening these connections among stakeholders and decision-makers will foster a more holistic and effective approach to sustainable development in the trainers’ industry.

## Electronic supplementary material

Below is the link to the electronic supplementary material.

Supplementary Material 1

## Data Availability

The data used for this study is publicly available on the following brands’ websites: https://tinyurl.com/29vjznfa, https://tinyurl.com/5n6pf2ur, https://shop.ethletic.com/en/, https://tinyurl.com/yc5nxkb9, https://orbashoes.eco/, https://sansmatin.co.uk/, https://eu.saola.com/, https://www.solerebels.com/, https://www.thousandfell.com/, https://www.waes.co/, https://tinyurl.com/2p9umv55, https://www.zeta-shoes.com/en-en, https://www.veja-store.com/en_uk/The data was collected between May 22nd and May 29 th, 2024.
