# Supplementary material for: An analysis of sustainability and performance indicators in Eco-Conscious trainers’ brands
Source: Sci Rep. 2025 Jul 1;15:21581. doi: 10.1038/s41598-025-04186-y (PMC12219566; doi:10.1038/s41598-025-04186-y)
Supplement: Supplementary file 1 — Supplementary Material 1 [file 41598_2025_4186_MOESM1_ESM.pdf]

Appendix

Code Book

**Code Name**	**Definition**	**Inclusion Criteria**	**Exclusion Criteria**	**Examples**
Environmental Sphere	Recognizes the actions that prevent, reduce or mitigate the ecological degradation.	Used when the description refers to a direct positive impact to the environment.	Exclude when the description represents a direct positive impact to the employees or the brand.	"By using recycled cotton, we extend the life of a fibre that has already been created."
Sustainable Materials	Encompasses materials which purpose is to reduce the environmental footprint of goods, include materials that come from certified, organic or regenerative natural or recycled sources, materials that are bio-based or biodegradable and alternative materials to petroleum-based compounds.	Used when the description refers to any material that comes from a certified, organic or regenerative natural source, has a percentage of recycled content, includes bio-based or bio-degradable compounds or acts as a natural, less harmful alternative to petroleum-based materials.	Exclude if the composition of the material is 100% virgin non-recyclable petroleum-based or if it comes from a non-certified, organic or regenerative source.	"Our footbed is 72% corn-based foam"
Natural Materials	Includes textile fibres that come from a certified, organic or regenerative natural source. Leather is included in this categorisation.	Used when the fibre's description follows the criteria of being certified, organic or regenerative.	Exclude if the material is not a textile fibre. Do not include if the material is combined with a petroleum-based components.	"Our laces are made from organic cotton"
Plant-based Materials	Refers to materials which composition comes mainly from a natural source and are not consider a textile fibre. Bio-based materials are included.	Used when the described material refers to a compound which total or major composition comes from nature.	Exclude if the material is a textile fibre.	"Our outsole is made from 65% natural rubber"

Recycled Materials	References materials which composition consist of a total, or majority, of reprocessed compounds.	Used when it is indicated that a material comes from reprocessed sources	Exclude if the material is indicated as virgin or as 100% natural	"The upper material consists of GRS-certified recycled PU leather.
Vegan Leather Alternatives	Encompasses leather-like materials that are being developed to substitute natural leather.	Applied when the description indicates that the material is an alternative to conventional leather.	Exclude when the description specifies natural leather. Do not include if the composite is not a leather-like material.	"From corn waste we have developed a vegan and bio-based material… friendly alternative to leather"
Other Components.	Comprises environmentally friendly materials used for additives, adhesives, coatings and finishings. Additionally, any other material that could not be included in the previous categorisations.	Applied when the described material served as a less harmful alternative to adhesives, coatings, finishings or relates to no other category in the study.	Exclude non-sustainable alternatives and bio-based materials that are not meant to be used as additives, adhesives, coatings or finishing.	"Water-based glue is used to laminate flax and ramie together for the outer"
Sustainable Manufacturing	Refers to production processes that aim to minimise the environmental impact of goods manufacturing. Sub-categorizations include local production, environmentally certified facilities, traditional techniques and production waste management	Applied when a manufacturing process description considers an alternative route to minimise the environmental footprint of the process. It is exclusively focused on the technique itself and not in the materials or design of the product.	Exclude if the described process is focused on the materials or the design stages.	"Our ethically handmade shoes"
Handcrafted Techniques	Encompasses traditional techniques that reduce the	Applied when the product has been made by handcraft or	Exclude if the manufacturing technique is not being specified or	"Assembled by hand in Portugal"

	reliance on machines and increase the quality and durability of the product.	ancient techniques.	indicated as handmade or handcraft.	
Local Production	Indicates a production made within a specific geographical area that relates to the brand's headquarters location and planned selling point.	Used when the production facilities are indicated to be within the same area in which the brand is based and the products are meant to be sell, location is specified.	Exclude when the manufacturing facilities location requires long transportations for products to be consumed and evaluated. Do not include if there is no location provided.	"First-ever made sneaker made in Los Angeles"
Recovery of Manufacturing Waste	Refers to the collection and reuse of the waste produced in the production processes.	Used when it is indicated that manufacturing residues are being used recollected and reused within the production stages.	Exclude when waste comes from the end of life of the product alone. Do not include if there is no indication of the material waste being reused.	"Reusable waste for insole manufacturing"
Environmentally Certified Manufacturing Facilities	References production factories that have received an accreditation for their positive environmental performance.	Used when it is specified that manufacturing facilities award a certification or accreditation related to their environmental impact.	Exclude if the certification or accreditation refers to good working conditions and fairtrade.	"All 4 of ISA TanTec tanneries are LWG Gold rated"
Sustainable Distribution	Comprises the overall logistics of the product but with a focus on reducing the environmental footprint on each stage. Considers environmentally conscious alternatives for packaging, reduced carbon	Used when the description refers to a low impact alternative to any step of the logistics stages.	Exclude when the description refers to the manufacturing stages or when the used alternative is not an environmentally friendly option	"All our shoeboxes and care cards are made from 100% recycled cardboard"

	emissions transportation and distribution.			
FSC certified Cardboard for packaging	Recognizes cardboard material packaging that comes from responsible and certified forests.	Used when the packaging description indicates that is certified By the Forest Stewardship Council (FSC).	Exclude if the packaging is described as reusable.	“All cardboard is recyclable, FSC certified, traceable”
Local Distribution	Identifies alternatives for the transportation of goods within a limited geographical area to reduce the carbon footprints caused by distribution.	Used when a green alternative to standard distribution services has been described for the transportation of the products.	Exclude if the transportation system requires to distribute products within long distances across different areas such as different countries.	“We are using bike messengers and local micro warehouse to deliver sneakers to our customers in NYC”
No Air Shipping	Refers to transportation methods that avoid air distribution to minimise the carbon emissions.	Used when the description states a no air shipment policy or when green distribution alternatives are being detailed.	Exclude when transportation is applied locally or when distances are reduced.	“We do not ship our products by air”
Biodegradable Packaging	References packaging aimed to naturally decompose without releasing toxic components, under a specific environment.	Applied when the described packaging is described as biodegradable.	Exclude when the packaging is described as FSC certified or reusable.	“All … packaging is biodegradable”
Recycled-Made Packaging	Encompasses packaging alternatives that are made from repurposed or reprocessed materials.	Applied when the packaging is being detailed as recycled or reprocessed.	Exclude if the packaging is described as reusable or biodegradable.	“Our packaging is made from 100% recycled paper”
Reusable Packaging	Includes alternative packaging options meant to be used multiple times before being discarded.	Applied when it is described that the packaging is designed to be reused.	Exclude it the packaging is defined as recycled or biodegradable.	“To wrap your sneakers in an organic cotton tote bag, please repurpose and reuse”

Renewable Energy supplied in the facilities.	Identifies logistic and corporate facilities that are powered by clean energy alternatives.	Applied when it is indicated that headquarters, warehouses, stores or any other logistic and administrative offices are being powered by green energy.	Exclude if the facilities described are related to the manufacturing stages of the product.	"Our two offices and four shops based in Paris, which have their electricity supplied by … 100% renewable energy"
End of Life Recovery	Encompasses the practices applied at the end of the product's life cycle to extend their lifetime and reclaim the materials to avoid them ending up in landfill. It considers recyclability, repairability and recovery.	Applied when a recovery method is being described to reclaim the materials at the end of the products life, or to extend their durability and use. Used when the scheme described is centred on the product alone.	Exclude when the information refers to the packaging of the product or any alternative components.	"A space thought as a laboratory to restore and recycle worn-out pairs"
Recyclability Schemes	Refers to initiatives applied to reclaim products at the end of their life cycle for the reprocessing and reintroduction of the materials on the supply chain.	Applied when the described program aims to retake the products for material's recovery.	Exclude when the description considers composting, biodegradation or any other natural process for the disposal of the material aside from recycling. Do not include if the program has not been developed yet.	"Complete the lifecycle of you old … sneakers by recycling them with us."
Repair Schemes	References the programs applied to ease the restoring and mending of products. These initiatives encourage consumers to extend products' lifetime.	Applied when the initiative's description indicates a program or physical location where consumers can have their products repaired.	Exclude when the initiative relies on recycling the product. Do not include if the program has not been applied yet.	"We have started offer our customers a discounted repair option… consumers can now extend the life span of their …. trainers."

Recovery Schemes for Composting	Recognizes products, components or materials that will break down under a defined time frame and natural circumstances at the end of their lifetime.	Applied when it is indicated that the product, component or material will decompose into nature under a composting environment.	Exclude if the description specifies biodegradation under controlled circumstances.	"Our shoes will be returnable for a credit note, ground down, composted and used as fertiliser"
Recovery schemes for Biodegradation	Identifies products, components or materials that will decompose under controlled circumstances at the end of their lifecycle.	Applied when the description indicates that the product, material or component will degrade under controlled circumstances to avoid landfill waste at the end of their lifetime.	Exclude if the description indicates that the product, component or material can be disposed on a composting environment.	"Shoes are returned to us … so we can make sure they are placed in the right microbial environment for biodegradation"
Eco-Design	Refers to the principles applied at the design stage of a product to plan its disposal and end of life recovery. It encompasses the disassembly, recyclability, longevity and durability of the product. These principles consider design alternatives alone, in contradiction to materials or manufacturing alternatives for the same purposes. Eco-design focuses on the solutions applied within a design level only.	Applied when the described alternatives are applied at a design level to increase recyclability, durability and longevity.	Exclude if the proposed alternatives are linked to the materials or the manufacturing techniques, even if they are aiming to the same purposes as Eco-Design.	"The footbed is designed to be easily removable which is important because it helps with cleaning and drying the shoe"
Design for Biodegradability	References products that were created to be	Applied when the design's description	Exclude when the design's description of the	"A casual sneaker that is

	biodegraded. Unlike materials that are meant to be biodegraded, this code acknowledges the product entirely.	indicates biodegradability as the end-of-life objective.	product refers to recyclability as the aim at the end of its life cycle.	designed to biodegrade"
Design for Durability	Identifies products that aim for longevity. Products that are created for staying in use for longer, extending their time frame. Centred on the design itself rather than the materials applied or the manufacturing techniques.	Applied when the design's description details that the product is meant to be used for longer due to its arrangement.	Exclude when the design's description of the product refers to its maintenance for durability.	"The goal of our design studio is simple: create sneakers that will stand the test of time."
Design for Easy Care	Encompasses the products that are built to be user-friendly care, minimising strict measures for cleaning.	Applied when the product's description indicates that its maintenance is simple and easy.	Exclude when the design's description of the product refers to an extended life cycle.	"Lasting sneakers that are easy to clean and refurbish"
Design for Recyclability	References products which design allows them to be reprocessed. Aiming for them to be recycled because of their construction.	Applied when the product's description denotes that its design principles applied on the product allows it to be reprocessed for recycling.	Exclude when the design's description of the product refers to recycled materials or components.	"Design into products that can be taken apart for recycling"
Design for Reducing Manufacturing Waste	Refers to products which assembly benefits the minimisation of manufacturing waste through the production processes. Unlike manufacturing techniques this code centres on the construction alone rather than	Applied when the product's design description recognizes its construction as the element that reduces waste along the manufacturing stages.	Exclude when the design's description of the product refers to the manufacturing technique alone and not to its construction or design.	"Knit construction avoids material waste"

	the process of making it.			
Economic Sphere	Identifies the actions that promote traceability in the supply chain and transparency in the internal operations related to profitability and assessment.	Used when the described actions allude to transparency in the internal processes and operations.	Exclude when the described actions allude to a direct positive impact towards the environmental or the employees.	“Customers can track where the raw materials come from, and in which certified companies the sneakers are manufactured”
Collaboration with Environmental Organizations	Indicates the alliance between the brand and NGOs that advocate for the Environmental Conservation.	Used when the description suggests an active partnership with organizations that promote the environment’s restoration and preservation.	Exclude when the organizations mentioned in the alliance promote standardised working conditions, fairtrade or research and education alone. Do not include if the alliance does not mention that a donation or contribution is made to the organisation.	“Started to work with Partnership for Forests (P4F) a British fund that supports forest conservation”
Collaboration with Health Organizations	Refers to the partnership linking the brand and NGOs committed to health research.	Used when an alliance with an organisation that centres in health and care research is indicated.	Exclude when the collaboration mentioned is made with NGOs that promote the environment’s preservation, fair working conditions or fairtrade alone. Do not include if the partnership does not mention that a contribution, or donation is made to the organisation.	“We partner with charities we feel resonate with us ……. The PoIG Foundation … mission is to support and accelerate research to find effective treatments and a cure for POLG
Collaboration with Social Organizations	Recognizes the cooperation between the brand	Used when a partnership is described	Exclude when the partnership is built with NGOs	“We partner with charities we feel

	and NGOs that advocate for society's wellbeing, bringing benefit to the community and communities.	indicating an active contribution to an organisation that supports the community welfare.	that promote environmental preservation, fair working conditions or education and research alone. Do not include if there is no indication of a contribution, or donation made to the organisation.	resonate with us ……. Children Change Colombia … working with children and their families to challenge poverty"
Collaboration with Work Organizations	References the alliance connecting brands with organizations that advocate to fairtrade, standardised working conditions and labour rights.	Used when an active partnership is indicated linking the brand with NGOs that promote fair labour rights.	Exclude when the alliance is built with organisations that promote environmental preservation, society's welfare, or education and research alone. Do not include if there is no indication of a contribution or donation made to the organisation.	"Funding training programs such as the International Labour Organisations Score Program to help them grow …. To future social compliance"
Educational Funds	Indicates the use of profit to finance education and research.	Used when it is indicated that educational or research activities are being funded through the brand.	Exclude when there is no statement that specifies that a contribution or donation is being used to fund education or research alone.	"We are proud to finance research activities on sustainability"
Monitoring of Supply Chains	Refers to the auditing and monitoring of the different stages across the manufacturing of goods.	Used when an audit or monitoring scheme is indicated generally and it does not specify a stage or process alone. Applied when supply chain stages can be traced.	Exclude when the monitoring or audit refers to the product alone or when it is indicated exclusively to one stage of the supply chain.	"Every year we perform a social audit to make sure our factories respect our values and meet our criteria"

Money Gender Gap	Refers to the monetary difference between male and female salaries.	Applied when actions or results related to the to the money gap between genders are being described.	Exclude when fair wages, fairtrade or fair living wage has been indicated alone and without a reference to gender.	"Money gender gap: 4 out of the 5 highest earners in the company are women."
Investment Initiatives	Encompasses the strategies related to the investment schemes within the brand.	Used when financing schemes or investment information from the business are indicated.	Exclude when the information refers to profit or payment schemes.	"We have chosen not to have investors to satisfy with quarterly dividends"
Traceability in Manufacturing Processes	Refers to the acknowledgment and disclosure of the manufacturing facilities' location and conditions.	Used when the circumstances, location or further information about the production facilities is being described and disclosed.	Exclude when the information disclosed refers to the raw material production facilities or the description indicates generally to the supply chain.	"Working close with long-experienced artisans located in Spain and Portugal"
Traceability in Raw Materials Production	Recognizes the capacity to track and disclose the raw material production facilities location and conditions.	Used when it is recognised and communicate the circumstances or location of the raw materials production facilities.	Exclude when the details refer to the manufacturing facilities of the product alone, or to a general indication of the conditions of the supply chain	"Cotton produced by 1,035 families in Brazil and Peru"
Transparency in the environmental impact	Encompasses data that reveals the environmental footprint of the manufacturing of goods.	Used when data about the environmental impact of the production is being publicly shared.	Exclude when the shared data is not related to the manufacturing of the goods. Do not apply if the information shared does not include numerical data.	"In 2020, SAOLA's carbon footprint is 130TCO_2eq all scopes combined"
Transparency in the Evaluation Internal System	Refers to the inner assessment self-developed tools to	Used when internal monitoring tools are being	Exclude when the monitoring tools cannot be	"Orba Flexible Framework Checklist"

	evaluate the brands' performance.	shared and disclosed publicly.	accessed or consulted. Do not include if the assessments are considering the environmental impact alone.	
Transparency in Price and Profit	Refers to the disclosure of financial information related to the profit made by the company in accordance with the selling of their products.	Used when information related to the profitable margin and financial benefits for the company is being indicated.	Exclude if the information provided encompasses a general financial report of the status of the company. Do not include if profitable margin description is not disclosed.	"We achieve a coefficient margin between 2 and 3"
Social Sphere	Encompasses the actions that directly represent an impact on peoples' life.	Used when the description illustrates a direct positive impact for the employees' rights and working conditions.	Exclude when the description illustrates a direct positive impact for the environment or the brand.	"Everybody who contributes to the creation of our sneakers performs their work in safe and ethical working conditions"
Fair Wages	References an equitable payment for employees accordingly to their working activities and standard living expenses.	Applied when it is expressed that employees are receiving a fair salary.	Exclude when there is no reference of an equitable payment or if independent payments are mentioned.	"Fair remuneration and decent work hours are just a few of the benefits of our people"
Incentives and Extra Bonuses	Refers to any independent payment apart from employees' salary, commonly given as a stimulus for improving workers' performance.	Applied when monetary stimuli are mentioned as a reward for employees' development and wellbeing.	Exclude when information refers to the employees' monthly payment.	"Providing full medical benefits and education funds for our employees"
Offers to Disable Workers	Recognizes working opportunities for disabled	Applied when it is described that disabled workers operate at any	Exclude when a diverse working environment is mentioned but	"In 2022, +200 employees recognized as disabled

	employees in the aim of increase inclusivity.	stage of the supply chain of trainers.	does not specify disabled employees within it.	workers were supported”
Paternity Leave	Identifies the opportunity for male workers to take a leave after his child is born.	Applied when the opportunity to take a leave due to a child’s birth is available for male employees.	Exclude when the description mentions maternity leave alone.	“3 months of paternity leave”
Regulated Working Conditions	References a balanced working environment with safe conditions, standardized working hours and rest schedules.	Applied when fair working conditions are mentioned along the stages of the supply chain.	Exclude when fair wages are mentioned alone and no further information in working conditions is provided.	“Are made exclusively in factories and workshops where International Labour Standards (ILO) are respected”
Social Indicators	Identifies standards that assess sustainability from a perspective centred on the wellbeing of workers and their rights.	Applied when the mentioned assessment tool refers to evaluating the wellbeing of employees and workers’ rights, assuring that these are being protected.	Exclude if the indicator assesses environmental and economic practices alongside or individually.	“Our company locations are certified… SA8000, the certification that ensures the protection of workers’ rights in the footwear production sector”
Economic Indicators	Refers to the assessment tools that evaluate sustainability from a perspective focused on the impact and growth of the financial decisions.	Applied when the described tool refers to the evaluation of the impact and growth of the economic activities of the business.	Exclude if the indicator assesses social and environmental practices alongside or individually	No references on this analysis were found for this code.
Environmental Indicators	Recognizes the assessment tools that evaluate sustainability from a perspective focused on the effects caused on the ecosystem. Refers to raw	Applied when the described standard refers to the evaluation of the environmental effects of the manufacturing of goods at the different stages.	Exclude if the indicator assesses social or economic practices alongside or individually.	“We have carried a life cycle analysis of our shoes in particular using the ADEME tool to understand their impact.”

	material certifications, manufacturing standards and green initiatives measuring tools.			
Economic and Environmental Indicators	References the standards that assess sustainability from a financial and ecological perspective alongside. Describes any assessment tool that considers both aspects within the same instrument.	Applied when the evaluation tool that has been described considers the assessment of the financial decisions and the environmental effects in parallel.	Exclude if the indicator assesses social practices alongside.	No references on this analysis were found for this code.
Economic and Social Indicators	Encompasses the assessment tools that evaluate sustainability considering workers' wellbeing and rights, and the economic growth of the company.	Applied when it is indicated that the financial decisions are evaluated alongside the workers' rights.	Exclude if the indicator assesses environmental practices alongside.	No references on this analysis were found for this code.
Environmental and Social Indicators	References the evaluation tools that assess sustainability considering the positive impact on workers' wellbeing and the ecosystem, alongside.	Applied when the described standard evaluates the impact on employees and the environment.	Exclude if the indicator assesses economic practices alongside.	"100% of our production facilities have signed and commit to respecting our ethical code …. Our code concerns our policies on the following topics … flexible working hours, … factories certified and powered by renewable energy"
Environmental, Social and	Refers to the assessment tools that evaluate	Applied when the description of the assessment tool	Exclude if the description of the indicator does	"Since 2004 we have been applying fair

Economic Indicators	sustainability regarding the impact on workers' wellbeing, the ecosystem and the financial decisions alongside. References the evaluation of the three spheres of the Triple Bottom Line	encompasses the three spheres of the TBL for their evaluation.	not clarity the assessment of the three spheres.	trade"<br><br>"The fairtrade Standards incorporate a holistic blend of social, economic and environmental criteria"
